# Body temperature-dependent microRNA expression analysis in rats: *rno-miR-374-5p* regulates apoptosis in skeletal muscle cells via *Mex3B* under hypothermia

**DOI:** 10.1038/s41598-020-71931-w

**Published:** 2020-09-22

**Authors:** Takahiro Umehara, Shinichiro Kagawa, Aiko Tomida, Takehiko Murase, Yuki Abe, Keita Shingu, Kazuya Ikematsu

**Affiliations:** 1 Division of Forensic Pathology and Science, Unit of Social Medicine, Course of Medical and Dental Sciences, Graduate School of Biomedical Sciences, Nagasaki University School of Medicine, 1-12-4 Sakamoto, Nagasaki, Nagasaki 852-8523 Japan; 2 Forensic Science Laboratory, Nagasaki Prefectural Police Headquarters, Nagasaki, Nagasaki Japan; 3 Division of Forensic Pathology and Science, Unit of Social Medicine, Course of Medical and Dental Sciences, Graduate School of Biomedical Sciences, Nagasaki University School of Medicine, Nagasaki, Nagasaki Japan

**Keywords:** Diagnostic markers, miRNAs

## Abstract

Forensic diagnosis of fatal hypothermia is considered difficult because there are no specific findings. Accordingly, exploration of novel fatal hypothermia-specific findings is important. To elucidate the molecular mechanism of homeostasis in hypothermia and identify novel molecular markers to inform the diagnosis of fatal hypothermia, we focused on microRNA expression in skeletal muscle, which plays a role in cold-induced thermogenesis in mammals. We generated rat models of mild, moderate, and severe hypothermia, and performed body temperature-dependent microRNA expression analysis of the iliopsoas muscle using microarray and quantitative real-time PCR (qRT-PCR). The results show that *rno*-*miR-374-5p* expression was significantly induced only by severe hypothermia. Luciferase reporter assay and qRT-PCR results indicated that *Mex3B* expression was regulated by *rno-miR-374-5p* and decreased with decreasing body temperature. Gene ontology analysis indicated the involvement of *Mex3B* in positive regulation of GTPase activity. siRNA analysis showed that *Mex3B* directly or indirectly regulated *Kras* expression in vitro*,* and significantly changed the expression of apoptosis-related genes and proteins. Collectively, these results indicate that *rno-miR-374-5p* was activated by a decrease in body temperature, whereby it contributed to cell survival by suppressing *Mex3B* and activating or inactivating *Kras*. Thus, *rno-miR-374-5p* is a potential supporting marker for the diagnosis of fatal hypothermia.

## Introduction

Diagnosis of fatal hypothermia is carried out based on a combination of several common findings, such as the difference in colour between blood from the right and left ventricles, Wischnewski’s spot, haemorrhage of iliopsoas muscle and so on, which are often observed in corpses exposed to cold^1–4^. However, these findings are also observed in other pathologies such as carbon monoxide poisoning, hydrocyanic acid poisoning, and stress^5–7^. In addition, it is possible that the above findings may be recognised when cold exposure and another cause of death compete, further complicating diagnosis. Accordingly, a specific diagnostic marker is necessary for accurate diagnosis of fatal hypothermia.

Various studies using biochemical, pathological, morphological, and molecular biological approaches in the adrenal gland^8^, pituitary gland^9–11^, kidney^12^, and serum^13,14^ have been performed to identify diagnostic markers of fatal hypothermia. We previously reported supporting markers for diagnostic of fatal hypothermia utilising mRNA of hypothalamus^15^ and iliopsoas muscle^16^. Importantly, as corpses are often found after a postmortem interval, examinations often do not represent the state at the time of death. Thus, when studying forensic samples, it is important to consider the effects of postmortem interval. mRNA is structurally unstable, and mRNA isolated from forensic samples is often degraded as a result of the postmortem interval. However, it has been reported that even highly degraded RNA can yield molecular information in postmortem tissues^17^, but with limitations. Although mRNA is relatively stable in a cold environment, a molecular marker that can withstand the postmortem interval is required. Recently, microRNA (miRNA) has been proposed as a molecule that can withstand the postmortem interval^18–20^. Therefore, we focused on body temperature-dependent miRNA expression in the iliopsoas muscle, which plays an important role in maintaining mammalian body temperature in a cold environment^21,22^. In this study, we used the hypothermic rat model we previously reported^16^, and performed body temperature-dependent miRNA expression analysis of the iliopsoas muscle using microarray.

The aim of this study was to elucidate the molecular mechanism in iliopsoas muscle during the course of fatal hypothermia, and identify useful molecular markers for the diagnosis of fatal hypothermia.

## Results

### Seventeen miRNAs were upregulated by severe hypothermia

**Figure 1:**
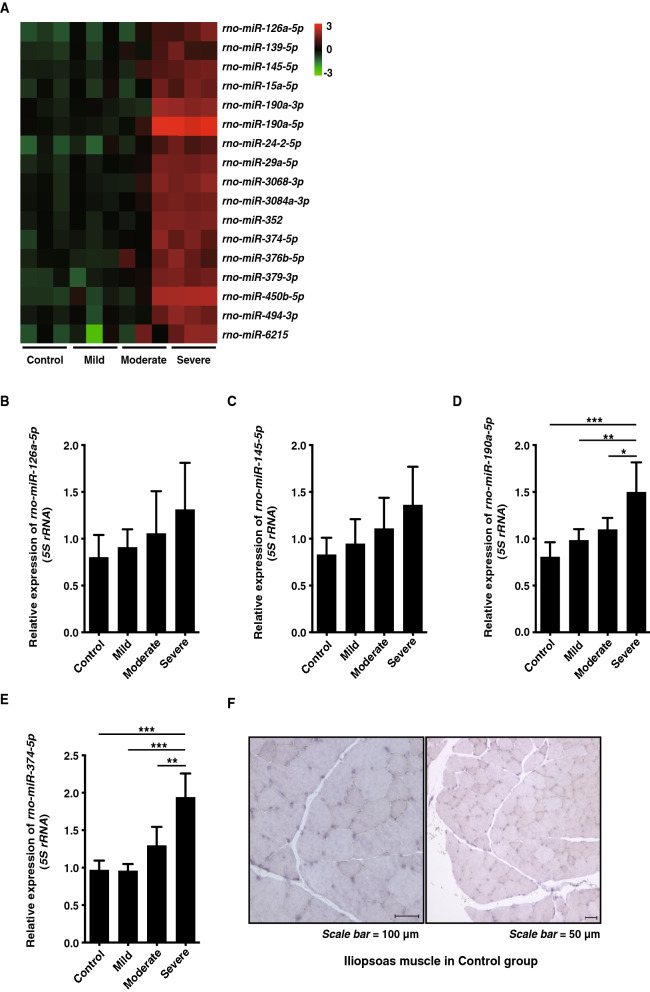
(**A**) Microarray analysis. A total of 17 miRNAs exhibited double the level of expression in severe hypothermia compared with the other groups. (**B**, **C**) Relative expression of *rno-miR-126a-5p* and -*miR-145-5p* in iliopsoas muscle. Both miRNAs were upregulated by a decrease in body temperature, but not significantly changed. (**D**, **E**) Relative expression of *rno-miR-190a-5p* and *rno-miR-374-5p* in iliopsoas muscle. Both miRNAs were significantly upregulated only by severe hypothermia. (**F**) In situ hybridisation showing localization of *rno-miR-374-5p* in iliopsoas muscle cells. Scale bar = 100 µm and 50 µm. Graphs show mean ± SD (n = 4–6). The statistical significance of differences between means was assessed by one-way ANOVA, followed by Tukey’s multiple comparison test. *P < 0.05, **P < 0.01, ***P < 0.001.

We performed a moderated *t* test (cut-off < 0.05) and Storey with bootstrapping for microarray data using GeneSpring. Microarray analysis showed that in the severe hypothermia group, expression levels of 17 miRNAs were more than doubled compared with control, mild, and moderate hypothermia groups (Fig. 1A, Table 1); whereas, levels of eight miRNAs in the severe hypothermia group were decreased to less than half compared with the other groups (Supplementary Table 1). Here, we focused on upregulated miRNAs. We predicted the mRNAs targeted by the 17 upregulated miRNAs using GeneSpring. Consequently, more than 100 mRNAs were extracted as target gene candidates (partial results are shown in Table 2). We focused on four miRNAs (*rno-miR-126a-5p, rno-miR-145-5p, rno-miR-190a-5p*, and *rno-miR-374-5p*) because the 100 target mRNAs were shown to be primarily controlled by these four miRNAs.

**Table 1:** Microarray fold-change analysis.

Symbol	Fold change (ctrl vs severe)	Regulation	Mirbase accession no.
*rno-miR-126a-5p*	2.8	Up	MIMAT0000831
*rno-miR-139-5p*	2.1	Up	MIMAT0000845
*rno-miR-145-5p*	2.3	Up	MIMAT0000851
*rno-miR-15a-5p*	2.3	Up	MIMAT0035728
*rno-miR-190a-3p*	2.7	Up	MIMAT0017145
*rno-miR-190a-5p*	5.4	Up	MIMAT0000865
*rno-miR-24–2-5p*	2.5	Up	MIMAT0005441
*rno-miR-29a-5p*	2.5	Up	MIMAT0004718
*rno-miR-3068-3p*	2.5	Up	MIMAT0024846
*rno-miR-3084a-3p*	2.3	Up	MIMAT0035740
*rno-miR-352*	2.4	Up	MIMAT0000610
*rno-miR-374-5p*	2.3	Up	MIMAT0003208
*rno-miR-376b-5p*	2.1	Up	MIMAT0003195
*rno-miR-379-3p*	2.6	Up	MIMAT0004791
*rno-miR-450b-5p*	4.4	Up	MIMAT0035746
*rno-miR-494-3p*	2.4	Up	MIMAT0003193
*rno-miR-6215*	3.1	Up	MIMAT0024854

Expression levels of 17 mirnas were more than doubled in severe hypothermia compared with control (Ctrl), mild, and moderate hypothermia.

**Table 2:** Predicted target genes of the four examined miRNAs.

GeneID	Mirbase accession no.	p-value	Symbol	Description	GO_ID
303,637	MIMAT0003208	0.02657807	Abca8	ATP binding cassette subfamily A member 8	GO:0,055,085|GO:0,043,231|GO:0,042,626|GO:0,016,021|GO:0,008,150|GO:0,006,869|GO:0,005,743|GO:0,005,575|GO:0,005,524|GO:0,003,674
81,732	MIMAT0003193,MIMAT0000865,MIMAT0000851	0.00707241	Actr3	ARP3 actin related protein 3 homolog	GO:2,000,251|GO:1,905,837|GO:1,905,835|GO:1,904,171|GO:1,903,078|GO:0,090,314|GO:0,071,560|GO:0,071,503|GO:0,071,364|GO:0,071,356|GO:0,071,347|GO:0,071,346|GO:0,061,851|GO:0,061,843|GO:0,061,832|GO:0,061,831|GO:0,061,830|GO:0,061,828|GO:0,061,825|GO:0,061,003|GO:0,060,271|GO:0,060,076|GO:0,051,965|GO:0,051,653|GO:0,051,491|GO:0,051,321|GO:0,051,117|GO:0,051,015|GO:0,050,775|GO:0,048,711|GO:0,048,708|GO:0,048,471|GO:0,045,666|GO:0,043,519|GO:0,035,984|GO:0,034,314|GO:0,033,206|GO:0,032,355|GO:0,032,092|GO:0,031,252|GO:0,030,838|GO:0,030,517|GO:0,030,056|GO:0,030,027|GO:0,016,344|GO:0,010,763|GO:0,010,592|GO:0,008,356|GO:0,007,283|GO:0,007,163|GO:0,005,911|GO:0,005,903|GO:0,005,885|GO:0,005,884|GO:0,005,737|GO:0,005,524|GO:0,005,515|GO:0,005,200|GO:0,003,779|GO:0,002,102|GO:0,001,726|GO:0,000,139
297,388	MIMAT0003208	0.02657807	Bola3	bolA family member 3	GO:0,008,150|GO:0,005,739|GO:0,005,575|GO:0,003,674
289,400	MIMAT0003193,MIMAT0003208,MIMAT0000851	0.0048565	Camsap2	calmodulin regulated spectrin-associated protein family, member 2	GO:1,990,752|GO:1,903,358|GO:0,061,564|GO:0,051,011|GO:0,050,773|GO:0,036,449|GO:0,033,043|GO:0,031,122|GO:0,031,113|GO:0,030,507|GO:0,007,026|GO:0,005,829|GO:0,005,813|GO:0,005,794|GO:0,005,516|GO:0,000,226
493,810	MIMAT0003208,MIMAT0035728	0.02504902	Capza2	capping actin protein of muscle Z-line alpha subunit 2	GO:0,051,016|GO:0,030,863|GO:0,016,020|GO:0,008,290|GO:0,005,903|GO:0,003,779
114,121	MIMAT0003208,MIMAT0000851	0.01217382	Ccnl1	cyclin L1	GO:1,901,409|GO:0,045,944|GO:0,045,737|GO:0,016,607|GO:0,016,538|GO:0,006,396|GO:0,006,351|GO:0,005,634|GO:0,005,515|GO:0,000,307
362,638	MIMAT0003208	0.02657807	Cda	cytidine deaminase	GO:0,071,217|GO:0,051,289|GO:0,046,898|GO:0,045,980|GO:0,042,803|GO:0,042,802|GO:0,030,308|GO:0,009,972|GO:0,008,270|GO:0,005,829|GO:0,004,126|GO:0,001,882
312,974	MIMAT0035728,MIMAT0000865,MIMAT0000845,MIMAT0003208	0.03259601	Chd7	chromodomain helicase DNA binding protein 7	GO:1,990,841|GO:0,060,429|GO:0,060,411|GO:0,060,384|GO:0,060,324|GO:0,060,173|GO:0,060,123|GO:0,060,041|GO:0,060,021|GO:0,050,890|GO:0,050,767|GO:0,048,844|GO:0,048,806|GO:0,048,771|GO:0,048,752|GO:0,045,944|GO:0,043,584|GO:0,043,010|GO:0,042,472|GO:0,042,471|GO:0,042,048|GO:0,040,018|GO:0,036,302|GO:0,035,909|GO:0,035,904|GO:0,035,116|GO:0,030,540|GO:0,030,217|GO:0,021,772|GO:0,021,553|GO:0,021,545|GO:0,016,817|GO:0,010,880|GO:0,008,150|GO:0,008,015|GO:0,007,628|GO:0,007,626|GO:0,007,605|GO:0,007,512|GO:0,007,417|GO:0,006,338|GO:0,005,730|GO:0,005,654|GO:0,005,634|GO:0,005,575|GO:0,005,524|GO:0,003,674|GO:0,003,226|GO:0,003,222|GO:0,003,007|GO:0,001,974|GO:0,001,701|GO:0,001,568|GO:0,001,501|GO:0,000,978
304,073	MIMAT0003208	0.02657807	Cldn14	claudin 14	GO:0,016,021|GO:0,008,150|GO:0,005,923|GO:0,005,886|GO:0,005,575|GO:0,005,198|GO:0,003,674
298,744	MIMAT0035728,MIMAT0003208,MIMAT0000851	0.04974522	Crim1	cysteine rich transmembrane BMP regulator 1	GO:0,030,165|GO:0,016,021|GO:0,010,951|GO:0,005,520|GO:0,004,867|GO:0,001,558
83,824	MIMAT0003193,MIMAT0000865	0.02504902	Cxxc4	CXXC finger protein 4	GO:0,031,410|GO:0,030,178|GO:0,030,165|GO:0,016,055|GO:0,008,270|GO:0,008,150|GO:0,005,829|GO:0,005,737|GO:0,005,634|GO:0,005,575|GO:0,005,515|GO:0,003,677
306,096	MIMAT0035728,MIMAT0003208,MIMAT0000851	0.02949709	Dach1	dachshund family transcription factor 1	GO:2,000,279|GO:0,060,244|GO:0,051,123|GO:0,048,147|GO:0,046,545|GO:0,045,892|GO:0,043,231|GO:0,033,262|GO:0,030,336|GO:0,010,944|GO:0,008,283|GO:0,007,585|GO:0,007,275|GO:0,006,355|GO:0,005,737|GO:0,005,667|GO:0,005,654|GO:0,005,634|GO:0,003,700|GO:0,001,967|GO:0,001,078|GO:0,001,075|GO:0,000,122
315,987	MIMAT0003208	0.02657807	Dcaf1	DDB1 and CUL4 associated factor 1	GO:1,990,245|GO:1,990,244|GO:0,080,008|GO:0,035,212|GO:0,033,151|GO:0,030,331|GO:0,030,183|GO:0,016,567|GO:0,008,180|GO:0,005,634|GO:0,001,650|GO:0,000,122
297,865	MIMAT0003193,MIMAT0035728,MIMAT0003208	0.01500606	Dsel	dermatan sulfate epimerase-like	GO:0,047,757|GO:0,030,205|GO:0,030,204|GO:0,016,021|GO:0,008,146
25,022	MIMAT0003193,MIMAT0003208	0.0302034	Fgfr2	fibroblast growth factor receptor 2	GO:0,090,263|GO:0,071,560|GO:0,071,300|GO:0,070,374|GO:0,070,372|GO:0,070,307|GO:0,061,031|GO:0,060,916|GO:0,060,915|GO:0,060,688|GO:0,060,687|GO:0,060,670|GO:0,060,667|GO:0,060,664|GO:0,060,615|GO:0,060,601|GO:0,060,595|GO:0,060,529|GO:0,060,527|GO:0,060,523|GO:0,060,512|GO:0,060,501|GO:0,060,484|GO:0,060,463|GO:0,060,449|GO:0,060,445|GO:0,060,442|GO:0,060,441|GO:0,060,365|GO:0,060,349|GO:0,060,348|GO:0,060,174|GO:0,060,076|GO:0,060,045|GO:0,055,010|GO:0,051,781|GO:0,051,150|GO:0,050,680|GO:0,050,679|GO:0,050,678|GO:0,050,673|GO:0,048,762|GO:0,048,755|GO:0,048,730|GO:0,048,701|GO:0,048,661|GO:0,048,608|GO:0,048,568|GO:0,048,565|GO:0,048,562|GO:0,048,557|GO:0,048,489|GO:0,048,333|GO:0,048,286|GO:0,046,777|GO:0,045,944|GO:0,045,839|GO:0,045,787|GO:0,045,471|GO:0,045,165|GO:0,044,344|GO:0,043,410|GO:0,043,235|GO:0,043,231|GO:0,043,066|GO:0,042,803|GO:0,042,802|GO:0,042,476|GO:0,042,472|GO:0,042,127|GO:0,042,060|GO:0,040,036|GO:0,040,014|GO:0,035,607|GO:0,035,604|GO:0,035,603|GO:0,035,602|GO:0,035,265|GO:0,035,264|GO:0,033,674|GO:0,032,808|GO:0,032,496|GO:0,031,434|GO:0,031,069|GO:0,031,012|GO:0,030,916|GO:0,030,901|GO:0,030,855|GO:0,030,324|GO:0,030,282|GO:0,030,177|GO:0,030,154|GO:0,022,612|GO:0,021,860|GO:0,021,847|GO:0,021,769|GO:0,018,108|GO:0,017,134|GO:0,016,331|GO:0,010,628|GO:0,010,518|GO:0,010,453|GO:0,009,986|GO:0,009,968|GO:0,009,887|GO:0,009,880|GO:0,009,791|GO:0,008,589|GO:0,008,543|GO:0,008,285|GO:0,008,284|GO:0,007,528|GO:0,007,417|GO:0,007,409|GO:0,007,275|GO:0,007,267|GO:0,005,938|GO:0,005,887|GO:0,005,737|GO:0,005,654|GO:0,005,634|GO:0,005,524|GO:0,005,007|GO:0,004,888|GO:0,004,871|GO:0,004,714|GO:0,004,713|GO:0,004,709|GO:0,003,416|GO:0,003,149|GO:0,003,148|GO:0,002,053|GO:0,001,837|GO:0,001,701|GO:0,001,657|GO:0,001,525|GO:0,000,122
64,845	MIMAT0000865,MIMAT0000851	0.04168744	Gphn	gephyrin	GO:0,099,634|GO:0,099,572|GO:0,098,970|GO:0,098,794|GO:0,097,112|GO:0,072,579|GO:0,061,599|GO:0,061,598|GO:0,060,077|GO:0,055,114|GO:0,051,260|GO:0,046,872|GO:0,045,211|GO:0,045,202|GO:0,045,184|GO:0,043,546|GO:0,043,025|GO:0,042,803|GO:0,032,947|GO:0,032,324|GO:0,031,234|GO:0,030,674|GO:0,030,425|GO:0,030,054|GO:0,018,315|GO:0,015,631|GO:0,010,038|GO:0,008,940|GO:0,007,529|GO:0,007,416|GO:0,006,777|GO:0,006,605|GO:0,005,856|GO:0,005,737|GO:0,005,622|GO:0,005,524|GO:0,005,515|GO:0,005,102
362,736	MIMAT0035728,MIMAT0003208,MIMAT0000851	0.00981946	Hectd1	HECT domain E3 ubiquitin protein ligase 1	GO:1,903,077|GO:0,070,534|GO:0,061,630|GO:0,060,708|GO:0,060,707|GO:0,051,865|GO:0,048,856|GO:0,046,872|GO:0,035,904|GO:0,030,154|GO:0,016,567|GO:0,005,737|GO:0,003,281|GO:0,003,170|GO:0,001,892|GO:0,001,843|GO:0,001,779
24,446	MIMAT0035728,MIMAT0003208,MIMAT0000865	0.01500606	Hgf	hepatocyte growth factor	GO:2,000,573|GO:1,902,947|GO:1,902,042|GO:1,901,299|GO:1,900,744|GO:0,090,201|GO:0,070,572|GO:0,061,138|GO:0,060,665|GO:0,060,326|GO:0,051,450|GO:0,050,918|GO:0,050,731|GO:0,050,728|GO:0,048,012|GO:0,046,982|GO:0,045,766|GO:0,043,154|GO:0,043,066|GO:0,042,802|GO:0,042,056|GO:0,035,729|GO:0,033,137|GO:0,032,733|GO:0,032,715|GO:0,031,643|GO:0,031,100|GO:0,030,335|GO:0,030,212|GO:0,014,068|GO:0,010,469|GO:0,008,284|GO:0,008,283|GO:0,008,083|GO:0,006,508|GO:0,005,615|GO:0,004,252|GO:0,001,934|GO:0,001,889|GO:0,000,902|GO:0,000,187
367,289	MIMAT0003208	0.02657807	Kansl1l	KAT8 regulatory NSL complex subunit 1-like	GO:0,046,972|GO:0,044,545|GO:0,043,996|GO:0,043,995|GO:0,035,035|GO:0,001,047|GO:0,000,123
259,273	MIMAT0000865,MIMAT0035728	0.0302034	Kcnq5	potassium voltage-gated channel subfamily Q member 5	GO:0,071,805|GO:0,030,118|GO:0,016,021|GO:0,008,076|GO:0,006,813|GO:0,005,887|GO:0,005,516|GO:0,005,251|GO:0,005,249
291,965	MIMAT0003208	0.02657807	Kctd19	potassium channel tetramerization domain containing 19	GO:0,051,260
361,029	MIMAT0000845,MIMAT0003208	0.0302034	Ktn1	kinectin 1	GO:0,045,296|GO:0,019,894|GO:0,016,021|GO:0,007,018|GO:0,005,783
300,866	MIMAT0003193,MIMAT0003208	0.02031241	Lca5	LCA5, lebercilin	GO:0,045,494|GO:0,042,073|GO:0,036,064|GO:0,015,031|GO:0,005,930|GO:0,005,929
361,028	MIMAT0003208,MIMAT0035728	0.03575591	Mapk1ip1l	mitogen-activated protein kinase 1 interacting protein 1-like	
308,790	MIMAT0000845,MIMAT0000865,MIMAT0003208	0.03246757	Mex3b	mex-3 RNA binding family member B	GO:0,072,697|GO:0,050,766|GO:0,043,547|GO:0,032,794|GO:0,022,409|GO:0,017,148|GO:0,005,829|GO:0,005,654|GO:0,003,727
361,605	MIMAT0000845,MIMAT0003208	0.02031241	Pcf11	PCF11 cleavage and polyadenylation factor subunit	GO:0,008,150|GO:0,006,379|GO:0,006,378|GO:0,006,369|GO:0,005,849|GO:0,005,739|GO:0,005,737|GO:0,005,654|GO:0,005,575|GO:0,003,729|GO:0,003,674|GO:0,000,993
307,401	MIMAT0003208	0.02657807	Pde6a	phosphodiesterase 6A	GO:0,060,041|GO:0,051,480|GO:0,047,555|GO:0,046,872|GO:0,046,037|GO:0,045,494|GO:0,008,150|GO:0,007,601|GO:0,007,165|GO:0,005,623|GO:0,005,575|GO:0,003,674
245,925	MIMAT0003208,MIMAT0000851	0.00183558	Phrf1	PHD and ring finger domains 1	GO:0,070,063|GO:0,046,872|GO:0,019,904|GO:0,006,397|GO:0,006,366
54,284	MIMAT0003193,MIMAT0003208	0.0302034	Pitx2	paired-like homeodomain 2	GO:2,000,288|GO:0,070,986|GO:0,061,325|GO:0,061,072|GO:0,061,031|GO:0,060,578|GO:0,060,577|GO:0,060,460|GO:0,060,412|GO:0,055,123|GO:0,055,015|GO:0,055,009|GO:0,055,007|GO:0,051,219|GO:0,048,738|GO:0,048,557|GO:0,048,536|GO:0,045,944|GO:0,045,893|GO:0,043,565|GO:0,043,388|GO:0,043,010|GO:0,042,803|GO:0,042,802|GO:0,042,476|GO:0,042,475|GO:0,042,127|GO:0,035,993|GO:0,035,886|GO:0,035,116|GO:0,033,189|GO:0,031,490|GO:0,031,076|GO:0,030,334|GO:0,030,324|GO:0,030,182|GO:0,021,983|GO:0,021,855|GO:0,021,763|GO:0,016,055|GO:0,009,887|GO:0,009,725|GO:0,009,653|GO:0,008,585|GO:0,008,584|GO:0,008,134|GO:0,007,520|GO:0,007,519|GO:0,007,507|GO:0,007,420|GO:0,007,368|GO:0,006,366|GO:0,006,357|GO:0,006,355|GO:0,005,737|GO:0,005,667|GO:0,005,634|GO:0,003,700|GO:0,003,682|GO:0,003,677|GO:0,003,350|GO:0,003,253|GO:0,003,171|GO:0,002,074|GO:0,001,764|GO:0,001,701|GO:0,001,570|GO:0,001,569|GO:0,001,191|GO:0,001,105|GO:0,001,102|GO:0,001,085|GO:0,001,078|GO:0,001,077|GO:0,000,981|GO:0,000,978|GO:0,000,976|GO:0,000,122
287,585	MIMAT0003193,MIMAT0035728,MIMAT0003208,MIMAT0000851	0.00177766	Ppm1d	protein phosphatase, Mg2 + /Mn2 + dependent, 1D	GO:0,051,019|GO:0,046,872|GO:0,045,814|GO:0,035,970|GO:0,009,617|GO:0,009,267|GO:0,006,468|GO:0,006,342|GO:0,006,306|GO:0,005,829|GO:0,005,634|GO:0,005,575|GO:0,004,722|GO:0,003,674|GO:0,000,086
81,762	MIMAT0003193,MIMAT0000845,MIMAT0000865,MIMAT0000831,MIMAT0000851	5.22E−04	Rock1	Rho-associated coiled-coil containing protein kinase 1	GO:2,000,114|GO:1,903,347|GO:1,903,140|GO:1,902,992|GO:1,902,430|GO:1,900,242|GO:1,900,223|GO:0,140,058|GO:0,106,003|GO:0,072,659|GO:0,051,894|GO:0,051,492|GO:0,051,451|GO:0,050,901|GO:0,050,900|GO:0,046,872|GO:0,045,664|GO:0,045,616|GO:0,043,524|GO:0,035,509|GO:0,032,970|GO:0,032,956|GO:0,032,091|GO:0,032,060|GO:0,032,059|GO:0,031,175|GO:0,030,866|GO:0,030,036|GO:0,030,027|GO:0,022,614|GO:0,017,049|GO:0,016,525|GO:0,010,628|GO:0,010,508|GO:0,007,266|GO:0,007,249|GO:0,007,159|GO:0,007,010|GO:0,006,915|GO:0,006,468|GO:0,005,886|GO:0,005,856|GO:0,005,814|GO:0,005,524|GO:0,004,674|GO:0,003,383|GO:0,001,726|GO:0,000,139
691,431	MIMAT0003208	0.02657807	Slc25a33	solute carrier family 25 member 33	GO:1,990,519|GO:1,990,314|GO:1,903,426|GO:0,071,156|GO:0,051,881|GO:0,034,551|GO:0,032,869|GO:0,031,966|GO:0,031,930|GO:0,030,307|GO:0,022,857|GO:0,016,021|GO:0,015,218|GO:0,008,284|GO:0,007,005|GO:0,006,864|GO:0,006,839|GO:0,006,390|GO:0,005,743|GO:0,002,082|GO:0,000,002
360,956	MIMAT0000865,MIMAT0000851	0.00881387	Tbc1d14	TBC1 domain family, member 14	GO:2,000,785|GO:1,902,017|GO:0,090,630|GO:0,071,955|GO:0,055,037|GO:0,043,231|GO:0,031,338|GO:0,019,901|GO:0,017,137|GO:0,010,507|GO:0,006,886|GO:0,005,829|GO:0,005,794|GO:0,005,776|GO:0,005,654|GO:0,005,096
294,595	MIMAT0003208	0.02657807	Ttc37	tetratricopeptide repeat domain 37	GO:0,055,087|GO:0,035,327|GO:0,005,829|GO:0,005,737|GO:0,005,654|GO:0,005,634
309,016	MIMAT0003208	0.02657807	Wdr11	WD repeat domain 11	GO:0,015,630|GO:0,008,150|GO:0,005,929|GO:0,005,829|GO:0,005,737|GO:0,005,575|GO:0,003,674
311,872	MIMAT0003193,MIMAT0035728,MIMAT0003208	0.04234872	Zbtb43	zinc finger and BTB domain containing 43	GO:0,003,676
311,718	MIMAT0035728,MIMAT0003208,MIMAT0000851	0.02403732	Zbtb46	zinc finger and BTB domain containing 46	GO:2,001,200|GO:2,001,199|GO:0,045,656|GO:0,045,650|GO:0,045,596|GO:0,030,853|GO:0,003,676
94,188	MIMAT0035728,MIMAT0003208,MIMAT0000851	0.03889311	Zfp423	zinc finger protein 423	GO:0,046,982|GO:0,046,872|GO:0,045,893|GO:0,045,892|GO:0,043,565|GO:0,042,803|GO:0,030,513|GO:0,030,154|GO:0,007,399|GO:0,007,219|GO:0,006,351|GO:0,005,654|GO:0,005,634|GO:0,003,676

### Severe hypothermia altered expression of *rno-miR-190a-5p* and *rno-miR-374-5p*

qRT-PCR using the SYBR Green I assay revealed that the expression of *rno-miR-126a-5p* and *rno-miR-145-5p* in iliopsoas muscle increased with decreasing body temperature, but did not change significantly (Fig. 1B,C). In contrast, expression of *rno-miR-190a-5p* and *rno-miR-374-5p* was significantly increased only by severe hypothermia compared with control, mild, and moderate hypothermia animals (Fig. 1D,E).

To determine which cells expressed *rno-miR-190a-5p* and *rno-miR-374-5p* in iliopsoas muscle, we performed in situ hybridisation (ISH). ISH results showed that *rno-miR-374-5p* was predominantly expressed in iliopsoas muscle cells (Fig. 1F), but *rno-miR-190a-5p* was not expressed (data not shown). Accordingly, we focused on *rno-miR-374-5p*.

### Expression of target mRNAs was altered with decreasing body temperature

**Figure 2:**
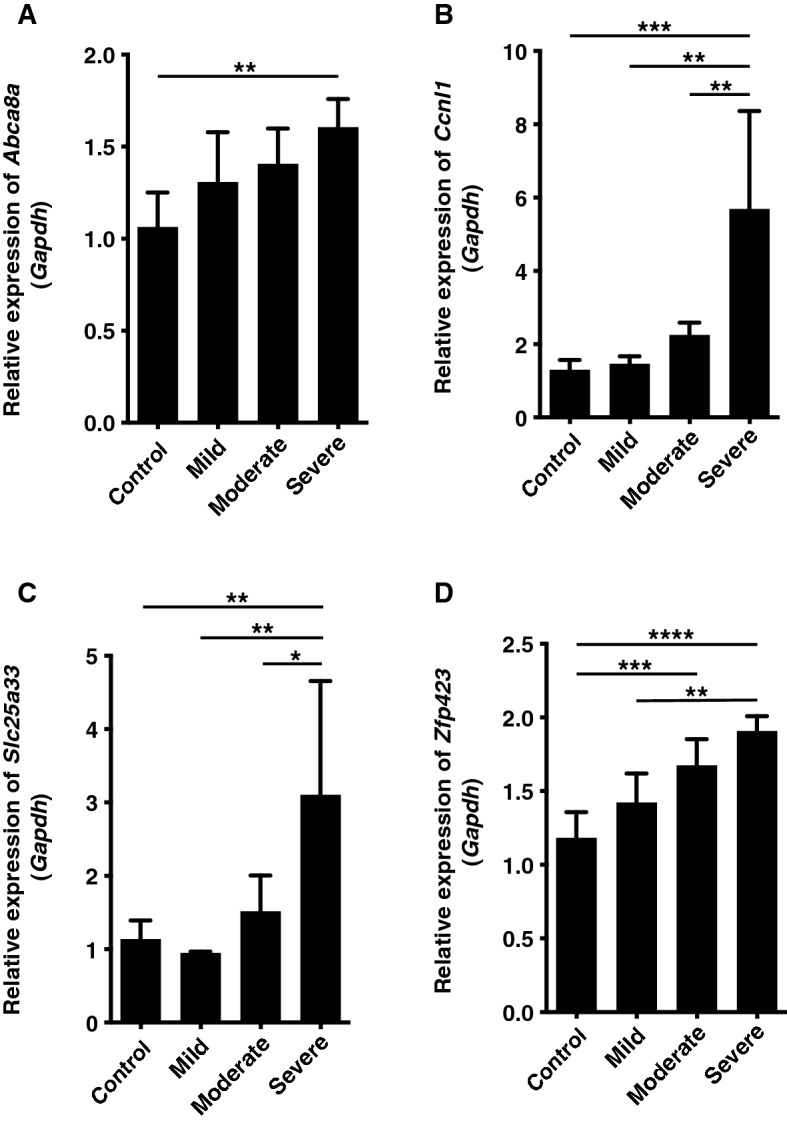
(**A**–**D**) Relative expression of *Abca8*, *Ccnl1*, *Slc25a33*, and *Zfp423* in each hypothermia group. These genes were induced by a decrease in body temperature. Expression levels were normalised to glyceraldehyde-3-phosphate dehydrogenase (*Gapdh*). Graphs show mean ± SD (n = 4–6). *P < 0.05, **P < 0.01, ***P < 0.001, ****P < 0.0001.

*Abca8a*, *Ccnl1*, *Slc25a33*, and *Zfp423*, which belong to “mitochondrial part”, “cell differentiation”, and “regulation of transcription” GO analysis terms (Table 2), are target gene candidates of *rno-miR-374-5p*. Expression of these genes was significantly altered with decreasing body temperature (Fig. 2A–D). In particular, expression of *Ccnl1* and *Slc25a33* was significantly increased only by severe hypothermia compared with control, mild, and moderate hypothermia animals.

Expression of *rno-miR-374-5p* was significantly increased only by severe hypothermia, suggesting that these genes might not be controlled by *rno-miR-374-5p* under hypothermic conditions. In our study, *rno-miR-30c-1-3p* was downregulated in the severe hypothermia group compared with the control group (Supplementary Table 1). TargetScan predicted *Slc25a33* as a target gene of *rno-miR-30c-1-3p* (https://www.targetscan.org/cgi-bin/targetscan/vert_72/view_gene.cgi?rs=ENST00000302692.6&taxid=10116&showcnc=0&shownc=0&shownc_nc=&showncf1=&showncf2=&subset=1). This result indicates that *Slc25a33* might be primarily regulated by *rno-miR-30c-1-3p* under hypothermic conditions. Accordingly, it is possible that in hypothermic conditions, factors such as other miRNAs promote the expression of these genes, and this mechanism is even more effective during severe hypothermic conditions. This issue will be a focus of future research.

### Expression of *Mex3B* exhibited an inverse correlation with *rno-miR-374-5p* expression

**Figure 3:**
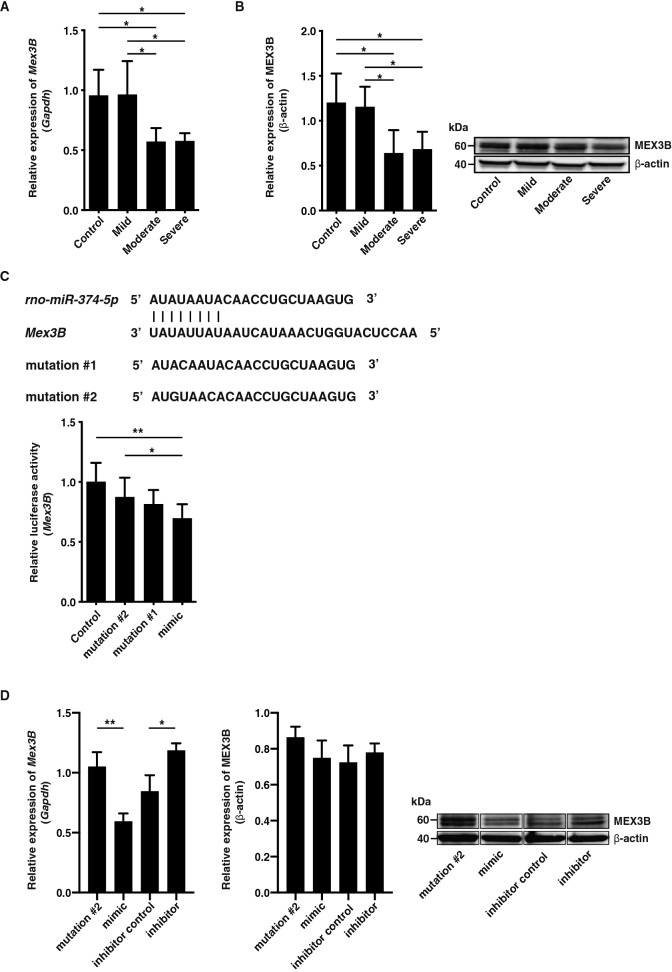
(**A**) Relative expression of *Mex3B* in each hypothermia group. These genes were induced by moderate and severe hypothermia. Expression levels were normalised to glyceraldehyde-3-phosphate dehydrogenase (*Gapdh*). (**B**) Relative level of MEX3B in each hypothermia group. Graphs show mean ± SD (n = 4–5). *P < 0.05. (**C**) Alignment of *rno-miR-374-5p* seed sequences and corresponding seed sequences of *Mex3B* mRNA. *rno-miR-374-5p* is predicted to bind with high affinity to the 3′-UTR of *Mex3B*. A luciferase reporter vector encoding the 3′-UTR was co-transfected with *rno-miR-374-5p* mimic or mutant into 3T3 cells. A decrease in luciferase activity indicated binding of the miRNA mimic to the 3′-UTR of the target sequence. Graphs show mean ± SD (n = 6–7). The statistical significance of differences between means was assessed by Mann–Whitney *U* test. *P < 0.05, **P < 0.01. (**D**) Relative expression of *Mex3B* and MEX3B in iliopsoas muscle cells transfected with *rno-miR-374-5p* mimic, mutation #2, *rno-miR-374-5p* inhibitor, or inhibitor control. Graphs show mean ± SD (n = 3). The statistical significance of differences between means was assessed by unpaired t test. *P < 0.05, **P < 0.01.

Expression of *Mex3B* was significantly decreased by moderate and severe hypothermia compared with control and mild hypothermia animals (Fig. 3A). In addition, the level of Mex3B protein after moderate and severe hypothermia was significantly decreased compared with the other groups (Fig. 3B). Expression of *rno-miR-374-5p* was gradually increased with decreasing body temperature, suggesting that this gene might be controlled by *rno-miR-374-5p* under hypothermic conditions.

### *rno-miR-374-5p* directly regulated *Mex3B* translation in vitro

To verify that the mRNA we identified were *bona fide* targets of *rno-miR-374-5p*, we employed a luciferase reporter assay, qRT-PCR, and western blotting. *rno-miR-374-5p* is predicted to bind with high affinity to *Mex3B*. In luciferase reporter assays, a decrease in luciferase activity indicates binding of the miRNA mimic to the 3′-UTR of the target sequence. Luciferase reporter assay, qRT-PCR, and western blotting results showed that the *rno-miR-374-5p* mimic could effectively inhibit *Mex3B* expression (Fig. 3C,D); thus, we concluded that *rno-miR-374-5p* directly regulates *Mex3B* expression in vitro.

### *rno-miR-374-5p* and *Mex3B* were involved in regulation of GTPase activity

**Figure 4:**
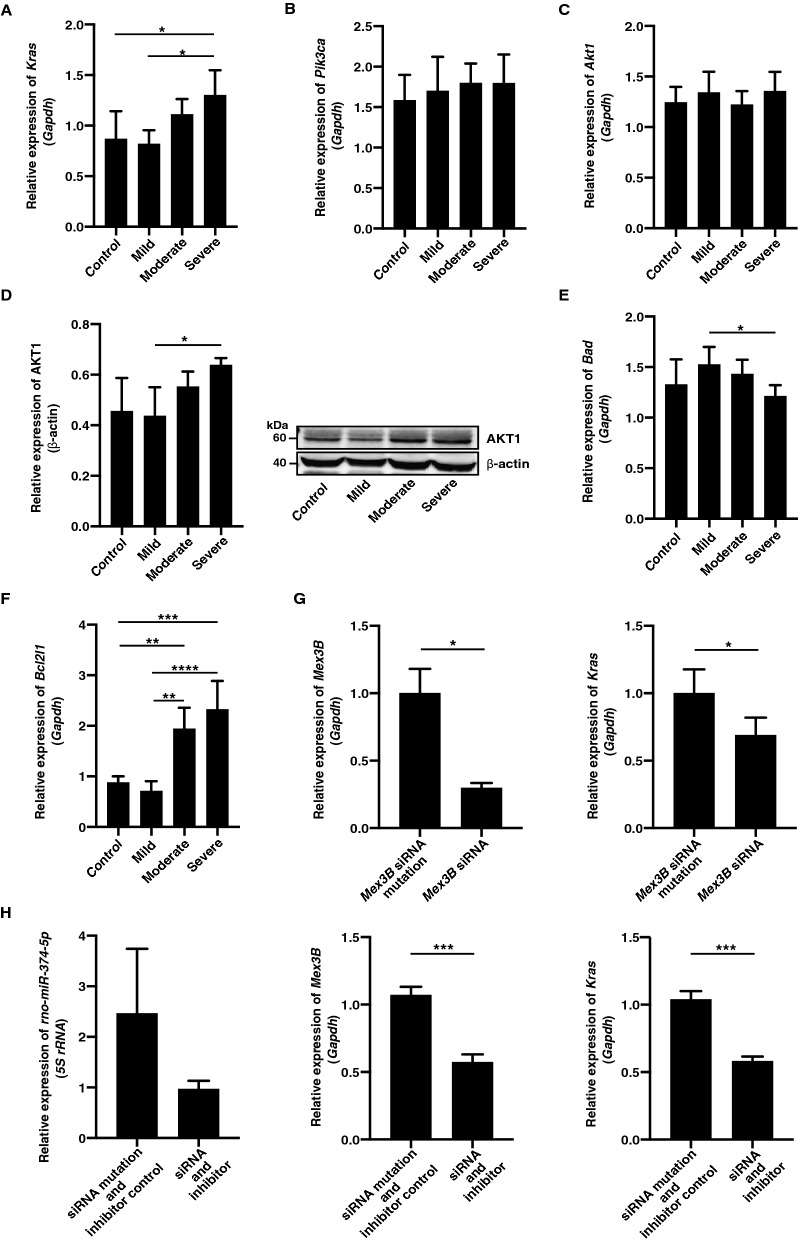
(**A**–**F**) Relative expression of *Kras*, *Pik3ca*, *Akt1*, AKT1, *Bad*, and *Bcl2l1* in each hypothermia group. Expression levels were normalised to glyceraldehyde-3-phosphate dehydrogenase (*Gapdh*) or β-actin. Graphs show mean ± SD (n = 3–6) (**G**) Relative expression of *Mex3B* and *Kras* between *Mex3B* siRNA and *Mex3B* siRNA mutant. Graphs show mean ± SD (n = 4). H. Relative expression of *rno-miR-374-5p*, *Mex3B,* and *Kras* between *rno-miR-374-5p* inhibitor and *Mex3B* siRNA and control. Graphs show mean ± SD (n = 3). The statistical significance of differences between means was assessed by unpaired t test. *P < 0.05, **P < 0.01, ***P < 0.001, ****P < 0.0001.

As *Mex3B* is involved in the induction of apoptosis by cellular stress and belongs to the GO term “positive regulation of GTPase activity” (Table 2), we focused on Ras, a GTPase that functions as a molecular switch for signalling pathways regulating cell survival, growth, and so on. We examined the expression of *Kras*, *Pik3ca*, *Akt1*, *Bad*, and *Bcl2l1*, which are involved in apoptosis according to the KEGG pathway. qRT-PCR using the SYBR Green I assay revealed that *Kras* expression in iliopsoas muscle increased with decreasing body temperature (Fig. 4A). Expression of *Pik3ca* was slightly increased with decreasing body temperature, but did not significantly change (Fig. 4B). Expression of *Akt1* was not significantly changed (Fig. 4C), but AKT1 expression significantly increased with decreasing body temperature (Fig. 4D). Expression of *Bad* significantly decreased with decreasing body temperature (Fig. 4E). In contrast, *Bcl2l1* expression significantly increased with decreasing body temperature (Fig. 4F).

To examine whether *Mex3B* regulated *Kras* expression, we synthesised siRNA for *Mex3B* and transfected iliopsoas muscle cells. As a result, *Mex3B* siRNA induced decreased *Mex3B* expression, followed by inactivation of *Kras* expression (Fig. 4G). This result contradicted observed *Kras* expression in the animal model. When we co-transfected iliopsoas muscle cells with the *rno-miR-374-5p* inhibitor and *Mex3B* siRNA, or transfected only *rno-miR-374-5p* inhibitor, *Kras* expression showed the same expression pattern as *Mex3B* (Figs. 3D, 4H and Supplementary Fig. 1A). However, when we transfected iliopsoas muscle cells with the *rno-miR-374-5p* mimic, *Mex3B* expression decreased and was followed by an increase in *Kras* expression (Fig. 3D and Supplementary Fig. 1B), consistent with the observed expression pattern in the animal model. Accordingly, these results suggest that *Kras* expression might be regulated not only by *Mex3B*, but also indirectly by activated *rno-miR-374-5p* in iliopsoas muscle cells.

### *rno-miR-374-5p* was involved in regulation of apoptosis in iliopsoas muscle cells

**Figure 5:**
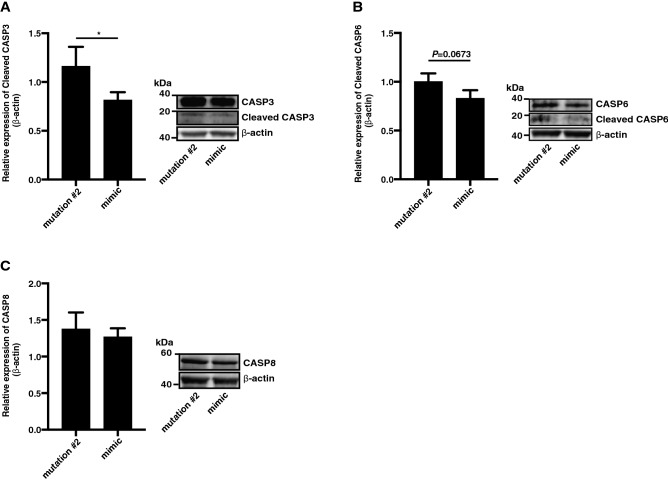
(**A**–**C**) Relative expression level of CASP3, CASP6, and CASP8 in iliopsoas muscle cells transfected with *rno-miR-374-5p* mimic, mutation #2, *rno-miR-374-5p* inhibitor, or inhibitor control. Graphs show mean ± SD (n = 3). The statistical significance of differences between means was assessed by unpaired t test. *P < 0.05.

Western blotting results revealed that overexpression of *rno-miR-374-5p* induced decreased activation of cleaved CASP3 and CASP6 in iliopsoas muscle cells compared with cells overexpressing *rno-miR-374-5p* mutant #2 (Fig. 5A,B). In addition, expression of CASP8 was slightly suppressed by overexpression of *rno-miR-374-5p* (Fig. 5C). These results indicate that activation of *rno-miR-374-5p* might enhance the viability of iliopsoas muscle cells.

## Discussion

In forensic practice, various biochemical, pathological, and molecular biological examinations are performed using blood and tissues collected from the corpse to assess the cause of death^8–16^. However, because corpses are often found with a postmortem interval, examinations often do not represent the state at the time of death. Accordingly, an index for diagnosis of cause of death that can withstand the postmortem interval is required. We have reported specific molecular markers using mRNA that support the diagnosis of fatal hypothermia, one of the pathologies for which the diagnosis of cause of death is difficult^15,16^. In a cold environment, mRNA is relatively stable, but a molecular marker that can withstand the postmortem interval is required. Recently, microRNA (miRNA) has been proposed as a molecule that can withstand the postmortem interval^18^. The iliopsoas muscle performs shivering- or non-shivering-thermogenesis to maintain body temperature when the body temperature declines extremely^23^, and haemorrhage of the iliopsoas muscle is observed in fatal hypothermia^3^. In our previous study, the iliopsoas muscle might have been activated because haemorrhage was observed in this muscle of rats with extreme hypothermia^16^. Therefore, we focused on miRNA expression in the iliopsoas muscle.

In this study, expression of *rno-miR-190a-5p* and *rno-miR-374-5p* in iliopsoas muscle was increased with decreasing body temperature. Previous reports suggest that the expression of both miRNAs fluctuates dynamically in response to hypoxia^24,25^. Hypothermic conditions induce tissue asphyxiation because blood is cooled by the inhalation of low-temperature air, and oxygen and hemoglobin do not dissociate in a cold environment. Therefore, both miRNAs may be activated by tissue asphyxiation induced by severe hypothermia and, therefore, be potential markers of hypoxia in a cold environment.

The expression of target gene candidates (*Abca8a*, *Ccnl1*, *Slc25a33* and *Zfp423*) of *rno-miR-374-5p* increased with decreasing body temperature and showed similar expression to *rno-miR-374-5p*. These results indicate that these genes may not be regulated by *rno-miR-374-5p*. *Abca8* is a sinusoidal efflux transporter for cholesterol and taurocholate in mouse and human liver^26^, but is function in hypothermia is still unknown. *Zfp423* is involved in skeletal muscle regeneration and proliferation after injury^27^. Increased expression of *Zfp423* may contribute the promotion of iliopsoas muscle repair. Expression of *Ccnl1* and *Slc25a33* was only increased by severe hypothermia. Inhibited expression of *Ccnl1*, a cell cycle regulatory gene, by forced expression of a specific miRNA suppressed cell proliferation and invasion, arrested cell cycle progression, and promoted cell apoptosis^28^. Hence, *Ccnl1* may contribute to cell survival during cellular stress such as cold stimulation. *Slc25a33* is reportedly involved in mitochondrial oxidative phosphorylation for ATP synthesis^29^. Accordingly, it is suggested that *Slc25a33* expression might be induced by the promotion of thermogenesis accompanying a decrease in body temperature. Moreover, *Ccnl1* and *Slc25a33* represent novel findings that will potentially complement the diagnosis of fatal hypothermia because the expression of both genes was induced only by severe hypothermia.

Our study showed that significantly decreased *Mex3B* expression in moderate and severe hypothermia was directly regulated by *rno-miR-374-5p*. *Mex3B* is reportedly involved in the induction of apoptosis following cell stresses such as heat, cold shock, reactive oxygen species, and radiation^30^. Accordingly, overexpression of *miR-374b-5p* inhibited apoptosis and contributed to the elevation of cell survival^31^. Our results indicate that *Mex3B* directly or indirectly regulated *Kras* expression, which was accompanied by subsequent activation of downstream molecules such as *Bcl2l1* in the Ras signalling pathway. *Kras* is a GTPase that functions as a molecular switch for signalling pathways regulating cell proliferation, survival, growth, and differentiation^32^. Bcl-2 family proteins regulate apoptosis through protein–protein interactions. Previous reports suggest that overexpression of Bcl‐2 inhibits apoptosis and plays an important role in the development of inflammation-related disorders^33,34^. Accordingly, these results suggest activation of a novel *rno-miR-374-5p*/*Mex3B*/*Kras* pathway to elevate cell viability in extreme hypothermia.

In conclusion, our results indicate that *rno-miR-190a-5p* and *rno-miR-374-5p* may be activated by tissue asphyxiation induced by severe hypothermia and, thus, serve as potential markers of hypoxia in a cold environment. In addition, *Ccnl1* and *Slc25a33* may be upregulated by cellular stresses such as cold shock and the promotion of thermogenesis accompanying decreases in body temperature. Thus, these miRNAs and mRNAs are potential novel supporting markers for the diagnosis of fatal hypothermia. In addition, a novel *rno-miR-374-5p*/*Mex3B*/*Kras* pathway may be involved in the pathological process of fatal hypothermia. However, further investigation of these genes is necessary before they can be applied to forensic practice.

## Materials and methods

All methods were performed according to relevant guidelines and regulation.

The Animal Care Committee of Nagasaki University approved this research protocol (approval number 1606081312-2).

### Animals

Pathogen-free 8-week-old male Wistar rats (300–350 g in body weight) were obtained from Charles River Laboratories (Yokohama, Japan). Prior to experiments, rats were housed for 1 week under a 12/l2-h light/dark cycle (light on at 07:00 and off at 19:00) at a constant temperature and humidity, and allowed free access to food and water.

### Experimental groups and thermal treatments

Twenty-three 9-week-old male Wistar rats were divided into four groups of 5–6 rats (control, mild, moderate, and severe hypothermia) and anesthetised by intraperitoneal injection of 0.3 mg/kg medetomidine, 2 mg/kg midazolam, and 2.5 mg/kg butorphanol. After 30 min, rats were exposed to cold (ambient temperature of 4 °C); rectal temperature (Ret) was continuously measured. Control rats were euthanised by cervical dislocation at 30 min after anesthesia. Mild hypothermia rats were euthanised by cervical dislocation when Ret reached 30 °C. Similarly, moderate and severe hypothermia rats were euthanised when Ret reached 22 °C and 12 °C, respectively^16^.

### RNA isolation and evaluation of total RNA integrity

After euthanasia, the iliopsoas muscle was immediately dissected, immersed in Ambion RNAlater (Thermo Fisher Scientific, Waltham, MA), and stored overnight at 4 °C. Total RNA, including microRNA (miRNA), was extracted using a miRNeasy Mini kit and miRNeasy Micro kit (Qiagen, Hilden, Germany) according to the manufacturer’s instructions. RNA samples were stored at − 80 °C until use.

Total RNA purity was assessed using a Nano-Drop2000 spectrophotometer (Thermo Fisher Scientific). RNA integrity was assessed by on-chip capillary electrophoresis using an RNA 6000 Nano kit and Agilent 2100 Bioanalyzer (Agilent Technologies, Santa Clara, CA). RNA integrity number was calculated as described elsewhere^35^.

### Microarray analysis

Microarray analysis was performed on a total of 12 samples (n = 3 per group) using a Rat miRNA V21.0 microarray in accordance with the manufacturer’s instructions (Agilent Technologies). Bioinformatic analyses were performed using GeneSpring v13 (Agilent Technologies). The data discussed in this publication have been deposited in NCBI’s Gene Expression Omnibus and are accessible through GEO Series accession number GSE139446.

### cDNA synthesis from miRNA and mRNA, and quantitative real-time PCR

Total RNA (10 ng) was utilised as a template for complementary DNA (cDNA) synthesis and quantitative real-time PCR (qRT-PCR), which were performed using a miRCURY LNA RT Kit and miRCURY LNA SYBR Green PCR Kit for miRNA expression analysis (Qiagen), respectively. Primers [*hsa-*(*rno-*)*miR-126-5p*, *hsa-*(*rno-*)*miR-145-5p*, *hsa-*(*rno-*)*miR-190a-5p*, *hsa-*(*rno-*)*miR-374b-5p,* and *5S rRNA*] were purchased from Qiagen.

Total RNA (500 ng) was utilised as a template for cDNA synthesis using a PrimeScript RT Reagent Kit for mRNA expression analysis (Takara Bio, Kusatsu, Japan) in accordance with the manufacturer’s instructions. qRT-PCR was performed in a 10-µL reaction system using SYBR Premix Ex Taq (Takara Bio) and a Thermal Cycler Dice Real-Time System (Takara Bio). Contents of the amplification mix and thermal cycling conditions were set in accordance with the manufacturer’s instructions. Primers [ATP binding cassette subfamily A member 8 (*Abca8a*), cyclin L1 (*Ccnl1*), solute carrier family 25 member 33 (*Slc25a33*), zinc finger protein 423 (*Zfp423*), Mex-3 RNA binding family member B (*Mex3B*), Kras proto-oncogene GTPase (*Kras*), phosphatidylinositol-4,5-bisphosphate 3-kinase*,* catalytic subunit alpha (*Pik3ca*), Akt serine/threonine kinase 1 (*Akt1*), Bcl2-associated agonist of cell death (*Bad*), apoptosis regulator Bcl-X (*Bcl2l1*) and glyceraldehyde-3-phosphate dehydrogenase (*Gapdh*)] were purchased from Takara Bio.

Relative quantification of miRNA and mRNA transcripts was performed using the ∆∆Ct method^36^.

### Total protein extraction and western immunoblot analysis

Iliopsoas muscles were homogenised using a TissueLyzer II (Qiagen). T-PER Reagent (Thermo Fisher Scientific), consisting of proteinase and dephosphorylation inhibitors, was then added. Debris was removed from the supernatant using an Ultrafree-MC 0.45-mm filter (Merck Millipore, Darmstadt, Germany). Filtered protein samples were quantified using a Direct Detect Spectrometer (Merck Millipore), separated on 4–12% NuPAGE Novex Bis–Tris gels (Thermo Fisher Scientific), transferred to polyvinylidene difluoride membranes, and blotted according to standard protocols (antibody details are listed in Table 3). Protein bands were visualised using ImmunoStar LD (Wako, Osaka, Japan), and band intensity was calculated using Multi Gauge version 3.X (Fujifilm, Tokyo, Japan). This method was performed according to our previous study^37^.

**Table 3:** List of antibodies.

Primary antibody (cat no.)	Species	Dilution	Blocking (manufacturer)	Secondary antibody (manufacturer)	Dilution
MEX3B (GTX32049)	Rabbit	1:1,000	PVDF blocking reagent (TOYOBO)	Anti-rabbit IgG HRP-linked whole antibody (GE healthcare)	1:50,000
AKT1 (ab126811)	Rabbit	1:1,000	PVDF blocking reagent (TOYOBO)	Anti-rabbit IgG HRP-linked whole antibody (GE healthcare)	1:100,000
CASP3 (ab13847)	Rabbit	1:500	PVDF blocking reagent (TOYOBO)	Anti-rabbit IgG HRP-linked whole antibody (GE healthcare)	1:100,000
CASP6 (CST#9762)	Rabbit	1:500	PVDF blocking reagent (TOYOBO)	Anti-rabbit IgG HRP-linked whole antibody (GE healthcare)	1:100,000
CASP8 (ab25901)	Rabbit	1:500	PVDF blocking reagent (TOYOBO)	Anti-rabbit IgG HRP-linked whole antibody (GE healthcare)	1:100,000
β-actin (GTX109639)	Rabbit	1:1,000	PVDF blocking reagent (TOYOBO)	Anti-rabbit IgG HRP-linked whole antibody (GE Healthcare)	1:100,000

### Synthesis of DNA, miRNA mimic, inhibitor, siRNA, and mutant

Putative target genes of *rno-miR-374-5p* were predicted using GeneSpring (Agilent Technologies). DNA synthesis of *Mex3B* was performed by Hokkaido System Science (Sapporo, Japan). Luciferase reporter plasmids were constructed to confirm the regulation of target genes by *rno-miR-374-5p*. As a negative control, a *rno-miR-374-5p* mimic (chemically synthesised double-stranded mature *rno-miR-374-5p*) and mutant were chemically synthesised by GeneDesign (Ibaraki, Osaka, Japan). Similarly, inhibitor and siRNAs for *rno-miR-374-5p*, *Mex3B* and a mutant negative control were chemically synthesised by GeneDesign.

### Cell culture and reagents

3T3 cells were cultured for the luciferase reporter assay in Dulbecco’s Modified Eagle’s Medium (DMEM, Wako) with high glucose, l-glutamine, 10% foetal bovine serum (FBS), and 1% penicillin–streptomycin. Cells were harvested, seeded onto a 96-well plate at a density of about 2.2 × 10^4^ cells per well in DMEM (Wako) with 10% FBS, but without 1% penicillin–streptomycin, and cultured for 24 h. Subsequently, cells were washed with Opti-MEM (Thermo Fisher Scientific) and 100 µL of Opti-MEM was added to each well for incubation at 37 °C prior to transfection.

### Transfection and luciferase reporter assay

The 3′-untranslated regions (UTRs) of *rno-miR-374-5p* targets were predicted using microT-CDS in DIANA TOOLS (https://diana.imis.athena-innovation.gr/DianaTools/index.php?r=microT_CDS/index). Vectors were constructed with pmirGLO Dual-Luciferase miRNA Target Expression Vector (Promega, Madison, WI) in accordance with the manufacturer’s instructions. Primers consisting of the 3′-UTRs of predicted *rno-miR-374-5p* target sequences and appropriate restriction sites were synthesised, annealed, and cloned downstream of the firefly luciferase reporter (*luc2*) gene in pmirGLO. Sequences were as follows (upper- and lowercase letters indicate the 3′-UTR and restriction sites for PmeI and XbaI, respectively): *Mex3B* sense 5′-aaacAACCTCATGGTCAAATACTAATATTATATt-3′, and *Mex3B* antisense 5′-ctagaATATAATATTAGTATTTGACCATGAGGTTgttt-3′. Sequences of the *rno-miR-374-5p* mimic and mutant of the seed sequence (as a negative control) were as follows: *rno-miR-374-5p* mimic 5′-AUAUAAUACAACCUGCUAAGUG-3′, *rno-miR-374-5p* mutation #1 5′-AUACAAUACAACCUGCUAAGUG-3′, and *rno-miR-374-5p* mutation #2 5′-AUGUAACACAACCUGCUAAGUG-3′.

3T3 cells (2.2 × 10^4^ cells/100 µL) were co-transfected with the *rno-miR-374-5p* mimic or mutant, and a reporter plasmid containing the 3′-UTR of *Mex3B*. The *rno-miR-374-5p* mimic and mutant were added at a final concentration of 45 nM along with Lipofectamine3000 (Thermo Fisher Scientific). Luciferase activity was assessed 48 h after transfection using a Dual-Glo Luciferase Assay System (Promega) according to the manufacturer’s instructions.

### In situ hybridisation

In situ hybridisation (ISH) was performed using a microRNA ISH buffer set and miRCURY LNA Detection 5′- and 3′-DIG-labelled probes (Qiagen) in accordance with the manufacturer’s instructions. In brief, 4% paraformaldehyde perfusion-fixed tissues were embedded in paraffin. Six-micrometer-thick sections were deparaffinised and incubated with Proteinase K solution (DAKO, Glostrup, Denmark) for 10 min at 37 °C. After washing in phosphate-buffered saline, sections were dehydrated. Hybridisation was performed using 40 nM miRNA probe in microRNA ISH buffer (Qiagen) at 50 °C for 3 h. Sections were rinsed in 5 × SSC buffer at 50 °C for 5 min, twice with 1 × SSC buffer at 50 °C for 5 min, twice with 0.2 × SSC buffer at 50 °C for 5 min, and with 0.2 × SSC buffer at room temperature for 5 min. Sections were treated with blocking solution (Nacalai Tesque, Kyoto, Japan) for 15 min at room temperature and then incubated with an anti-DIG antibody (1:800; Roche Diagnostics, Basel, Switzerland) in blocking solution (Nacalai Tesque) overnight at 4 °C. Sections were developed using NTB/BCIP (Roche Diagnostics) at 30 °C. Observations were made using a BZ-9000 (Keyence, Osaka, Japan). The obtained images were processed with analysis software (Keyence). This method was performed according to our previous study^37^.

### Skeletal muscle cell culture and transfection

Rat iliopsoas muscle tissue was dissociated into a single-cell suspension using a skeletal muscle dissociation kit (Miltenyi Biotec, Bergisch Gladbach, Germany). For *rno-miR-374-5p* mimic or inhibitor or *Mex3B* siRNA assays, iliopsoas muscle cells were cultured in Skeletal Muscle Cell Growth Medium (SMCGM, Takara Bio). Cells were then harvested, seeded onto a six-well plate at a density of approximately 0.5–1.5 × 10^5^ cells per well in SMCGM, and cultured for 24 h. Subsequently, cells were washed with Opti-MEM, supplemented with 3 mL of Opti-MEM in each well, and incubated at 37 °C prior to transfection.

Iliopsoas muscle cells were transfected with *rno-miR-374-5p* mimic, mutation #2, *rno-miR-374-5p* inhibitor, inhibitor control, *Mex3B* siRNA, or mutant siRNA. *rno-miR-374-5p* inhibitor, inhibitor control, *Mex3B* siRNA, and mutant siRNA sequences were as follows (upper- and lowercase letters indicate RNA and DNA, respectively): *rno-miR-374-5p* inhibitor sense 5′-GACGGCGCUAGGAUCAUCAACCACUUAGCAGGUUGUAUUAUAUCAAGUAUUCUGGU-3′, *rno-miR-374-5p* inhibitor antisense 5′-ACCAGAAUACAACCACUUAGCAGGUUGUAUUAUAUCAAGAUGAUCCUAGCGCCGUC-3′, *rno-miR-374-5p* inhibitor control sense 5′-GACGGCGCUAGGAUCAUCAACUAUCGCGAGUAUCGACGUCGAGGCCCAAGUAUUCUGGU-3′, *rno-miR-374-5p* inhibitor control antisense 5′-ACCAGAAUACAACUAUCGCGAGUAUCGACGUCGAGGCCCAAGAUGAUCCUAGCGCCGUC-3′, *Mex3B* siRNA sense 5′-ACAGCAGACACAUACAUAUtt-3′, *Mex3B* siRNA antisense 5′-AUAUGUAUGUGUCUGCUGUtt-3′*, Mex3B* siRNA mutation sense 5′-ACAUCAGACACACACAUAUtt-3′*,* and *Mex3B* siRNA mutation antisense 5′-AUAUGUGUGUGUCUGAUGUtt-3′.

*rno-miR-374-5p* mimic, mutation #2, *rno-miR-374-5p* inhibitor, or inhibitor control were added at a final concentration of 45 nM along with Lipofectamine RNAiMAX Transfection Reagent (Thermo Fisher Scientific). *Mex3B* siRNA and mutant siRNA were used at 25 pmol/well with Lipofectamine RNAiMAX Transfection Reagent. Expression of *Mex3B*, *Kras*, MEX3B, CASP3, CASP6, and CASP8 was assessed 48 h after transfection by qRT-PCR and western blotting, in accordance with the manufacturer’s instructions.

### Data analysis

Data are shown as mean ± SD. Statistical significance of differences between means was assessed by Mann–Whitney *U* test, unpaired t test, and one-way ANOVA, followed by Tukey’s multiple comparisons test (GraphPad Software, San Diego, CA). A *P*-value < 0.05 was considered significant.

## Supplementary information

Supplementary Information.
