# Supplementary material for: Body temperature-dependent microRNA expression analysis in rats: rno-miR-374-5p regulates apoptosis in skeletal muscle cells via Mex3B under hypothermia
Source: Sci Rep. 2020 Sep 22;10:15432. doi: 10.1038/s41598-020-71931-w (PMC7508983; doi:10.1038/s41598-020-71931-w)
Supplement: Supplementary file 1 — Supplementary Information. [file 41598_2020_71931_MOESM1_ESM.pdf]

Supplementary materials

Body temperature-dependent microRNA expression analysis in rats: *rno-miR-374-5p* regulates apoptosis in skeletal muscle cells via *Mex3B* under hypothermia

Takahiro Umehara[1]*, Shinichiro Kagawa[1, 2], Aiko Tomida[3], Takehiko Murase[1], Yuki Abe[1], Keita Shingu[1], Kazuya Ikematsu[1]

Supplementary Table

Supplementary Table 1. Microarray fold-change analysis. Expression levels of eight miRNAs were decreased to less than half in severe hypothermia compare with control (Ctrl).

Symbol	Fold change (Ctrl vs Severe)	Regulation	mirbase accession No
rno-let-7c-1-3p	3.3	down	MIMAT0017087
rno-miR-203a-3p	3.0	down	MIMAT0000876
rno-miR-30c-1-3p	2.9	down	MIMAT0004719
rno-miR-3550	3.1	down	MIMAT0017808
rno-miR-3552	4.0	down	MIMAT0017813
rno-miR-3588	5.0	down	MIMAT0017887
rno-miR-433-5p	2.7	down	MIMAT0017192
rno-miR-671	10.2	down	MIMAT0005326

Supplementary Figure Legends

Supplementary Figure 1. Relative expression of *Kras* in iliopsoas muscle cells transfected with rno-*miR-374-5p* mimic, mutation #2, *rno-miR-374-5p* inhibitor or inhibitor control. Graphs show mean ± SD (n = 3). The statistical significance of differences between means was assessed by unpaired t test. $^{*}P < 0.05$.

Supplementary Figure 2. Full-length gels of western blots

Supplementary Figure 1

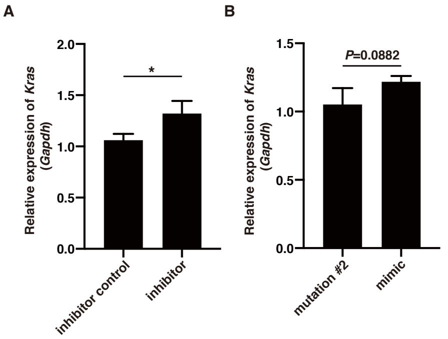

Supplementary Figure 2

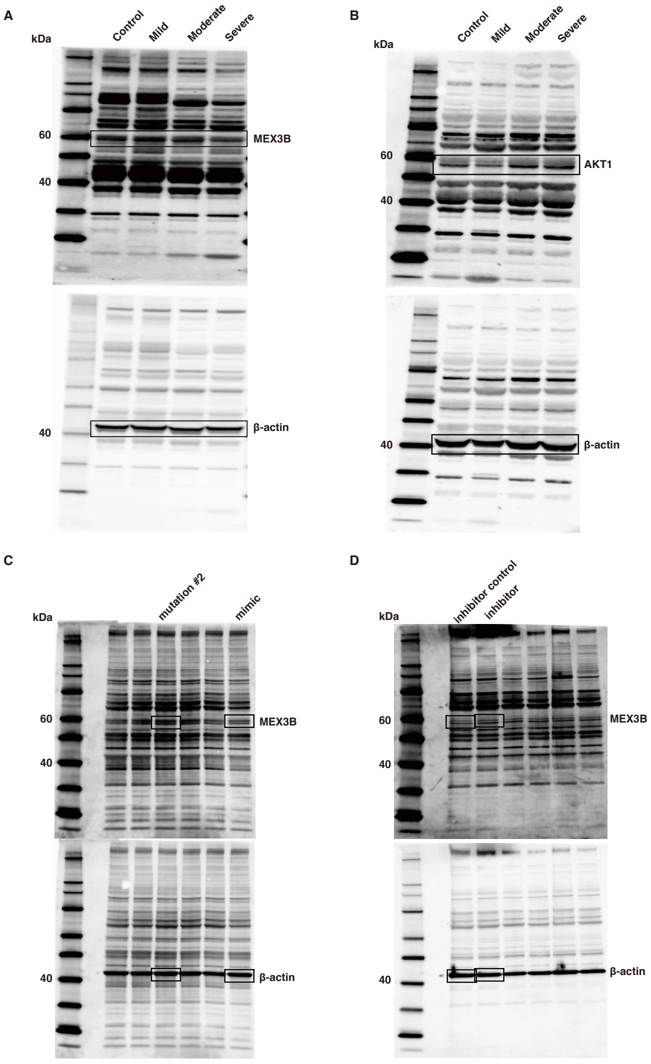

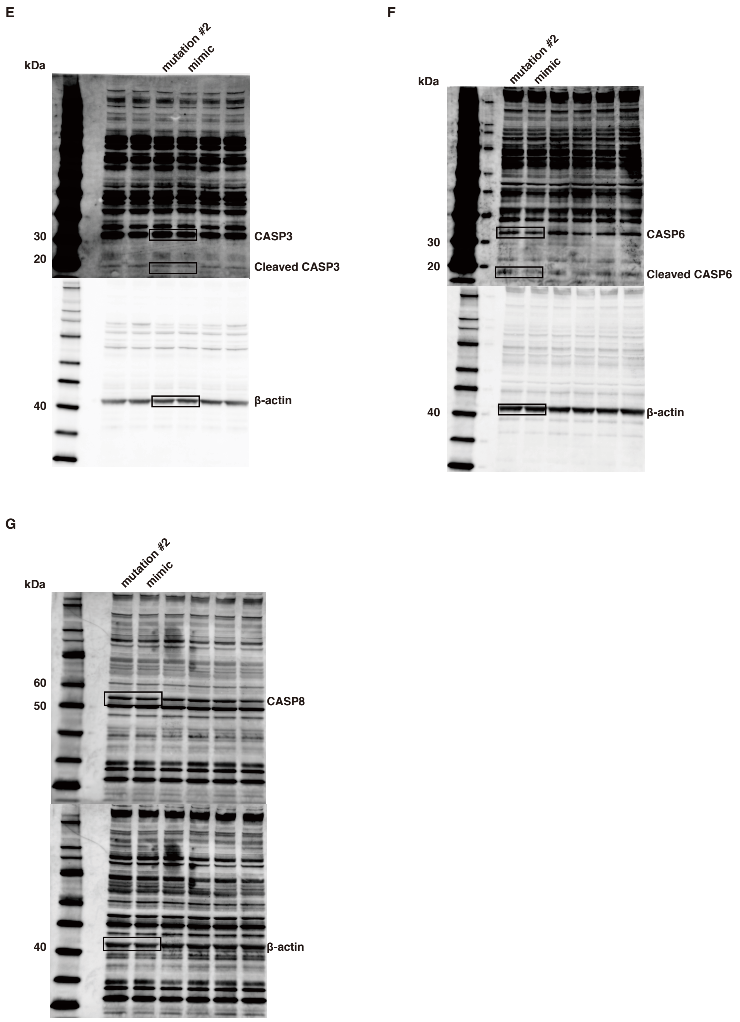
E
mutation #2
mimic
kDa
30
20
40
CASP3
Cleaved CASP3
β-actin
F
mutation #2
mimic
kDa
30
20
40
CASP6
Cleaved CASP6
β-actin
G
mutation #2
mimic
kDa
60
50
40
CASP8
β-actin
